# A Unique Immune-Related Gene Signature Represents Advanced Liver Fibrosis and Reveals Potential Therapeutic Targets

**DOI:** 10.3390/biomedicines10010180

**Published:** 2022-01-16

**Authors:** Pil-Soo Sung, Chang-Min Kim, Jung-Hoon Cha, Jin-Young Park, Yun-Suk Yu, Hee-Jung Wang, Jin-Kyeoung Kim, Si-Hyun Bae

**Affiliations:** 1 The Catholic University Liver Research Center, Department of Biomedicine & Health Sciences, College of Medicine, The Catholic University of Korea, Seoul 06591, Korea; pssung@catholic.ac.kr (P.-S.S.); chajh07@hanmail.net (J.-H.C.); 2 Division of Gastroenterology and Hepatology, Department of Internal Medicine, College of Medicine, Seoul St. Mary’s Hospital, The Catholic University of Korea, Seoul 06591, Korea; 3 CbsBioscience, Inc., Daejeon 34036, Korea; kcm3879@cbsbio.com (C.-M.K.); jonnypark@cbsbio.com (J.-Y.P.); yuyunsuk@cbsbio.com (Y.-S.Y.); 4 Department of Pharmacy, College of Pharmacy, CHA University, Seongnam 13488, Korea; kyeoung@cha.ac.kr; 5 Department of Surgery, Inje University Haeundae Paik Hospital, Busan 48108, Korea; wanghj@ajou.ac.kr; 6 Division of Gastroenterology and Hepatology, Department of Internal Medicine, College of Medicine, Eunpyeong St. Mary’s Hospital, The Catholic University of Korea, Seoul 03383, Korea

**Keywords:** gene signature, liver fibrosis, immune response, meta-analysis, macrophage

## Abstract

Innate and adaptive immune responses are critically associated with the progression of fibrosis in chronic liver diseases. In this study, we aim to identify a unique immune-related gene signature representing advanced liver fibrosis and to reveal potential therapeutic targets. Seventy-seven snap-frozen liver tissues with various chronic liver diseases at different fibrosis stages (1: *n* = 12, 2: *n* = 12, 3: *n* = 25, 4: *n* = 28) were subjected to expression analyses. Gene expression analysis was performed using the nCounter PanCancer Immune Profiling Panel (NanoString Technologies, Seattle, WA, USA). Biological meta-analysis was performed using the CBS Probe PINGS^TM^ (CbsBioscience, Daejeon, Korea). Using non-tumor tissues from surgically resected specimens, we identified the immune-related, five-gene signature (CHIT1_FCER1G_OSM_VEGFA_ZAP70) that reliably differentiated patients with low- (F1 and F2) and high-grade fibrosis (F3 and F4; accuracy = 94.8%, specificity = 91.7%, sensitivity = 96.23%). The signature was independent of all pathological and clinical features and was independently associated with high-grade fibrosis using multivariate analysis. Among these genes, the expression of inflammation-associated FCER1G, OSM, VEGFA, and ZAP70 was lower in high-grade fibrosis than in low-grade fibrosis, whereas CHIT1 expression, which is associated with fibrogenic activity of macrophages, was higher in high-grade fibrosis. Meta-analysis revealed that STAT3, a potential druggable target, highly interacts with the five-gene signature. Overall, we identified an immune gene signature that reliably predicts advanced fibrosis in chronic liver disease. This signature revealed potential immune therapeutic targets to ameliorate liver fibrosis.

## 1. Introduction

Chronic liver diseases progress to liver cirrhosis, which affects 1–2% of people worldwide. There is a high death rate associated with cirrhosis, and patients die due to cirrhosis-related complications, such as gastrointestinal bleeding or hepatocellular carcinoma (HCC), which develop in one-third of cirrhosis cases [1]. Several approaches to reduce liver fibrosis are under investigation in order to eradicate hepatic decompensation, HCC, and other fatal complications of cirrhosis [2], although no specific anti-fibrotic treatment is currently available. Recent strategies based on imaging and laboratory testing are not sufficiently predictive for identifying fibrotic progression and/or fibrogenic/carcinogenic activity in chronic liver diseases. Therefore, biomarkers with better reliability and sensitivity to predict liver fibrosis and patient outcomes are urgently required. Meanwhile, laboratory parameters, including the levels of serum albumin, bilirubin, and platelets, are within the normal range, and their smaller dynamic range is susceptible to non-specific variation, which makes them unreliable for predicting fibrosis progression or patient outcome [3].

A therapeutic target for anti-fibrosis may be revealed through expression analyses using liver biopsy specimens to distinguish between early and advanced liver fibrosis. A recently developed Nanostring nCounter technology makes it possible to perform gene expression analyses using minimal tissue samples [4]. The NanoString nCounter system is a nucleic acid hybridization platform that reliably detects the expression of up to 800 genes in a single assay [4]. Further biological meta-analyses make it possible to identify potential pathways and druggable targets after gene expression is measured [5]. These analyses may specifically focus on immune-related genes, which may be critically associated with liver fibrogenesis.

Intrahepatic macrophages are known to play critical roles in inflammation and fibrosis in chronic liver diseases [6]. Kupffer cells (KCs) are liver-resident, non-migratory macrophages capable of self-renewal and are derived from erythromyeloid progenitor cells in the fetal yolk sac [6,7]. They exist in the hepatic sinusoids and are different from the circulating monocyte-derived macrophages (MoMfs) originating from the bone marrow [8]. A recent study using livers from patients with nonalcoholic fatty liver disease showed that gut-derived LPS causes liver injury and inflammation by activating intrahepatic immune cells, such as KCs and MoMfs [9]. Activated macrophages then produce proinflammatory cytokines and amplify intrahepatic inflammation [10]. MoMfs differentiate into profibrogenic TREM2^+^CD9^+^ scar-associated macrophages and expand in the fibrotic liver when intrahepatic chronic inflammation is not stopped [11].

Overall, intrahepatic macrophages are critical for the initiation and progression of liver fibrosis. To identify potential therapeutic targets, in this study we perform a gene expression analysis using freshly frozen liver tissues at various stages of fibrosis using the nCounter PanCancer Immune Profiling Panel. Based on these analyses, we identified a macrophage-associated, novel immune-related gene signature associated with advanced fibrosis. Furthermore, we identified a potential druggable target derived from the gene signature and the resultant meta-analyses.

## 2. Methods

### 2.1. Patients and Tissue Samples

This study included 77 patients with histologically confirmed liver fibrosis. The inclusion criteria were as follows: (1) benign liver tissues with chronic liver diseases; (2) tissues obtained during hepatobiliary surgical procedures such as liver resection due to HCC. The exclusion criterion was the following: tissues that contain RNA with low purity or concentration not eligible to be analyzed by the nCounter PanCancer Immune Profiling Panel. Seventy-seven consecutive tissues were surgically obtained between 1995 and 2016 from the Ajou Medical Center. The retrospective protocol of this study was approved by the Institutional Review Board of the Ajou Medical Center (AJIRB-BMR-KSP-18-444) and The Catholic University of Korea (XC20EEDI0034). Fibrosis stage of the liver tissues were determined using the METAVIR scoring system.

### 2.2. RNA Extraction

RNA extraction was performed according to published methods [12]. Total RNA was extracted from non-tumor fibrosis tissues using a RNeasy Mini Kit (QIAGEN, Hilden, Germany) with DNase I treatment (QIAGEN). RNA quality was verified by RNA integrity using a Bioanalyzer 2100 (Agilent Technologies, Santa Clara, CA, USA). The total RNA concentration was measured using a Nanodrop 2000 (Thermo Fisher Scientific, Waltham, MA, USA).

### 2.3. Gene Expression Assay

Gene expression profiles were analyzed using nCounter MAX (NanoString Technologies, Seattle, WA, USA). The total reaction volume was 15 µL, containing 100 ng of RNA, reporter probes, and capture probes. The nCounter PanCancer Immune Profiling Panel (NanoString Technologies) was used for gene set profiling. Quality control and normalization of the raw data were performed using nSolver Analysis Software v4.0 (NanoString Technologies).

### 2.4. Gene Combination Analysis

**Figure 1:**
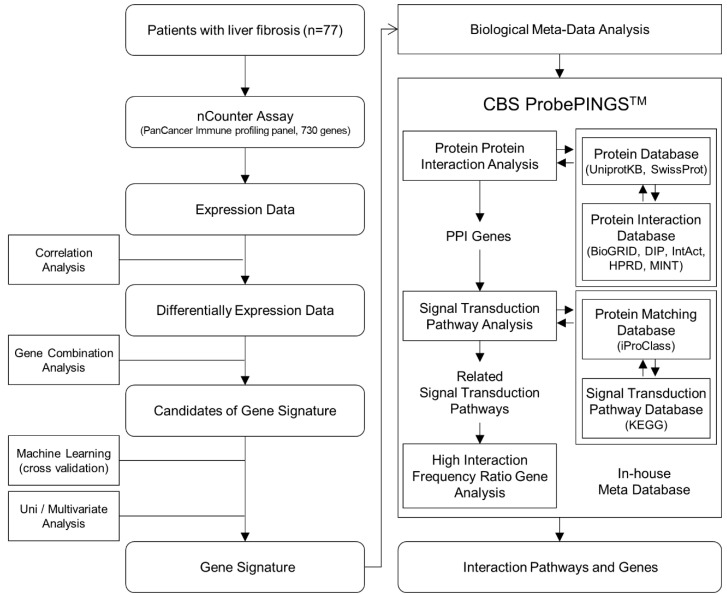
Flow chart of gene signature development and meta-analysis.

Figure 1 shows a flow chart of the gene signature development and meta-analysis. Differentially expressed genes were defined as those that were significantly differentially expressed more than 2-fold between fibrosis stages 1 and 2 and fibrosis stages 3 and 4. Of the differentially expressed genes, the genes that were statistically significant in logistic regression analysis were included in the gene combination analysis. For the combination of differentially expressed genes, we identified the logistic regression coefficient of each gene and weighted gene expression with the corresponding coefficient value.

### 2.5. Cross-Validation as Pre-Validation of Candidate Gene Signatures

The candidate gene signatures (AUC > 0.9, accuracy > 90%, *p* < 0.05) were pre-validated by k-fold cross-validation to identify the optimal gene combination. The patients were randomly separated by 2-folds (training set and test set) 300 times.

### 2.6. Biological Meta-Analysis

Biological meta-analysis was performed using the CBS Probe PINGS^TM^ (Reg. No. 2008-01-129-000568, CbsBioscience, Daejeon, Korea) that contains the following five modules: PPI, Path-Finder, Path-Linker, Path-Marker, and Path-Lister [5]. For gene signature validation, related signal transduction pathways were analyzed separately with the gene signature and differentially expressed genes of each fibrosis stage 3 and 4 patients. We selected the top 10 signal transduction pathways for the gene signature and each fibrosis stage 3 and 4 patients and identified highly interacting genes in these 10 pathways. We also compared the related gene signature pathways and all pathways related to fibrosis stage 3 and 4 patients. We compared the frequently interacting genes of the gene signature and all genes related to fibrosis stage 3 and 4 patients.

### 2.7. Statistical Analysis

The relationship between fibrosis stage and clinicopathologic variables was evaluated using the Chi-squared, Fisher’s exact, or Wilcoxon rank sum tests. Significant differences between fibrosis stages 1 and 2 and fibrosis stages 3 and 4 were evaluated using the Wilcoxon rank-sum test. Receiver operating characteristic curve analysis was used to determine the accuracy of threshold values for classifying fibrosis stages 1 and 2 and fibrosis stages 3 and 4. Gene signature independence was analyzed using logistic regression analysis between the gene signature and clinicopathologic variables. Statistical significance was set at *p* < 0.05 (two-tailed). All statistical analyses were performed in R version 3.4.3 (R Development Core Team, https://www.r-project.org/, accessed on 19 July 2021).

## 3. Results

### 3.1. Patient Characteristics

Baseline characteristics of the enrolled patients are presented in Table 1. This included patients with fibrosis stage 1 (*n* = 12), stage 2 (*n* = 12), stage 3 (*n* = 25), and stage 4 (*n* = 28). To identify the factors associated with advanced fibrosis (F3/4), we divided the enrolled patients into two groups: low- (F1/2, *n* = 24) and high-grade fibrosis (F3/4, *n* = 53). We defined low-grade and high-grade fibrosis according to the recent report describing significant worse liver-related outcome in patients with F3 and F4 fibrosis [13]. These benign fibrotic liver tissues were obtained from the non-tumor portion of the resected liver due to HCC. Therefore, the enrolled patients were predominantly male, and the principal etiology of chronic liver disease was chronic HBV infection (Table 1). In Korea, antiviral drugs for chronic HBV infection with normal liver enzymes are only reimbursed for patients with liver cirrhosis. Therefore, patients with advanced fibrosis were more likely to be treated with antiviral drugs. In most of the clinical and laboratory parameters, there were no statistically significant differences between the two groups, except for the platelet level, which was lower in patients with advanced liver fibrosis.

### 3.2. Expression Analysis-Derived Gene Signatures

**Figure 2:**
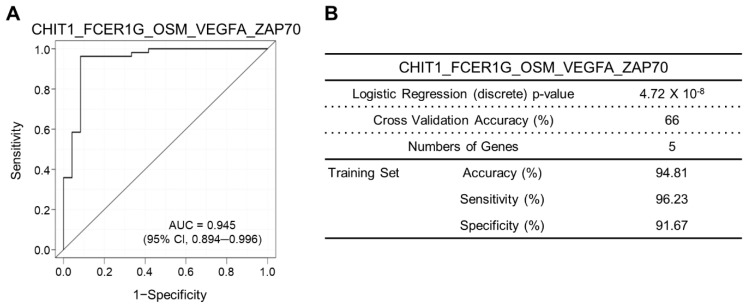
Clinical performance evaluation of the selected 5-gene signature. The clinical performance of the 5-gene signature was evaluated using receiver operating characteristic (ROC) analysis, cross-validation, and logistic regression analysis. (**A**) ROC analysis of 5-gene signature to the advanced fibrosis stage. (**B**) Clinical performance of the 5-gene signature in logistic regression analysis, cross-validation, and ROC analysis.

**Figure 3:**
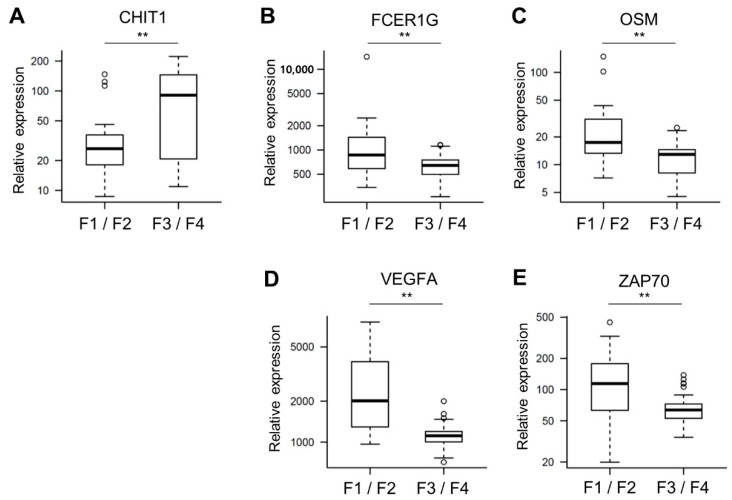
Expression of each gene comprising the gene signature in early stage versus late-stage liver fibrosis. Relative expressions of 5 genes in 24 patients with early-stage fibrosis and in 53 patients with late-stage fibrosis. ** *p* < 0.01. (**A**) CHIT1. (**B**) FCER1G. (**C**) OSM. (**D**) VEGFA. (**E**) ZAP70.

We developed immune-related gene signatures in our patient tissue samples using a NanoString nCounter PanCancer Immune Profiling Panel. Nine candidate gene signatures were identified (Table 2). The rank of each gene signature was determined using the AUC of the receiver operating characteristic curves and positive and negative predictive values. The immune-related gene signature that was composed of CHIT1_FCER1G_OSM_VEGFA_ZAP70 was demonstrated to be the most accurate gene signature for predicting F3/4 fibrosis in chronic liver diseases (Table 2). The AUC of the receiver operating characteristic (ROC) curve of CHIT1_FCER1G_OSM_VEGFA_ZAP70 was 0.945 (95% CI, 0.894–0.996; Figure 2), and the *p*-value by logistic regression analysis was 4.72 × 10^−8^. The sensitivity and specificity of the gene signature were 96.23% and 91.67%, respectively. The positive and negative predictive values were 96.23% and 91.67%, respectively. For the other expression-analyses-derived gene signatures, we confirmed that these gene signatures can also accurately predict advanced fibrosis (Table 2). Figure 3 depicts the expression of each gene comprising the most accurate gene signature in F1/2 and F3/4 fibrosis. Chitotriosidase (CHIT1) expression (Figure 3A) was increased in F3/4 fibrosis and that of FCER1G, OSM, VEGFA, and ZAP70 (Figure 3B–E) was decreased. Supplementary Figure S1 describes the expression of each gene comprising the gene signature in each stage of liver fibrosis.

We further performed the expression analysis comparing F0 liver tissues (*n* = 17) and fibrotic liver tissues (F1/2, *n* = 24, F3/4, *n* = 53) of chronic liver diseases by the same technique. The data are presented in the Supplementary Figure S2. There was no significant difference between F0 and F1/2 or F0 and F3/4 fibrosis (Supplementary Figure S2).

**Figure 4:**
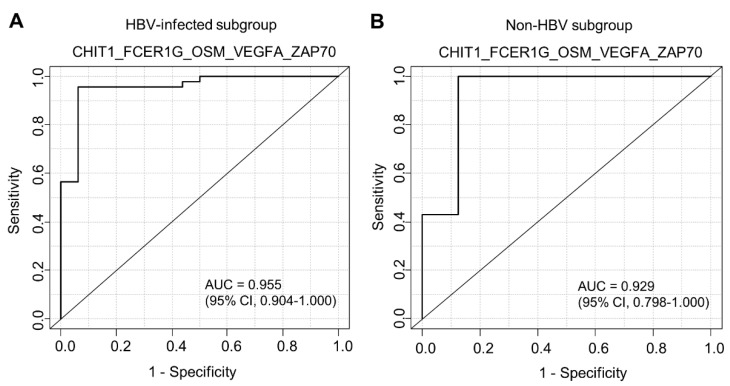
Expression of each gene comprising the gene signature in each stage of liver fibrosis in the HBV ((**A**), *n* = 62) and non-HBV ((**B**), *n* = 15) subgroups.

Next, we divided our cohort into two subgroups: HBV-infected (*n* = 62) and non-HBV patients (*n* = 15) (Figure 4). First, we validated our gene signature (CHIT1_FCER1G_OSM_VEGFA_ZAP70) in the HBV-infected subgroup (Figure 4A). In the HBV-infected subgroup, the AUC of the ROC curve of CHIT1_FCER1G_OSM_VEGFA_ZAP70 was 0.955 (95% CI, 0.904–1.000), and the *p*-value by logistic regression analysis was 4.72 × 10^−6^ (Figure 4A). In the non-HBV subgroup, the AUC of the ROC curve of CHIT1_FCER1G_OSM_VEGFA_ZAP70 was 0.929 (95% CI, 0.798–1.000), and the *p*-value by logistic regression analysis was 9.97 × 10^−1^ (not significant) (Figure 4B).

### 3.3. Factors Associated with Pathological High-Grade Fibrosis

Next, univariate and multivariate analyses were performed with the gene signature and clinical parameters in order to identify the factors associated with pathological high-grade fibrosis (Table 3). Gene signature, HBV infection, and platelet count were significantly associated with high-grade fibrosis in univariate analyses. These three statistically significant factors were selected as the parameters for multivariable analysis. Multivariate analysis revealed that only the gene signature was a statistically significant factor associated with high-grade fibrosis (odds ratio = 521.08, 95% CI: 31.21–8700.42, *p* < 0.001; Table 3).

### 3.4. Pathways and Genes Significantly Associated with the Gene Signature

Next, meta-analysis was performed to identify the pathways and genes significantly associated with the gene signature. The five-gene signature predictive of high-grade fibrosis was significantly associated with the Kyoto Encyclopedia of Genes and Genomes signal transduction pathways, including Kaposi’s sarcoma-associated herpesvirus infection, HPV infection, and EBV infection. The high interaction frequency genes associated with the gene signature were phosphoinositide-3-kinase regulatory subunit 1 (PIK3R1) and signal transducer and activator of transcription 3 (STAT3) (Table 4).

## 4. Discussion

In this study, we identified the immune gene signature that reliably predicts advanced fibrosis in chronic liver diseases using the nCounter PanCancer Immune Profiling Panel. This signature revealed potential immune therapeutic targets (STAT3) to ameliorate liver fibrosis.

Liver fibrosis is a common pathological consequence of most chronic liver diseases. Non-resolving liver injury is frequently accompanied by dysregulated wound healing and tissue repair, resulting in excessive deposition of the extracellular matrix. Intrahepatic macrophages play critical roles during this process, which can be reflected by the phenomenon that hepatic macrophage depletion alleviates liver fibrogenesis in mice [14,15]. In the early liver injury stage, KC CCL2 recruits proinflammatory and profibrogenic MoMFs, simultaneously producing proinflammatory cytokines to interact with hematopoietic stem cells to establish a profibrogenic niche [6,16,17]. A recent scRNA-seq study demonstrated a previously unidentified macrophage type in the fibrotic niche of the human liver [11]. TREM2^+^CD9^+^ scar-associated macrophages are monocyte-derived cells in the fibrotic liver and terminally differentiated, showing a pro-fibrogenic phenotype [11,18]. Moreover, LC3-associated phagocytosis was reported in patients with cirrhosis [14,19]. LC3-associated phagocytosis is a non-canonical autophagy that triggers a phenotype change of MoMfs to an anti-inflammatory phenotype [14,19]. The pharmacological inhibition of LC3-associated phagocytosis in monocytes has demonstrated that the FcγRIIA-mediated activation of the anti-inflammatory pathway is caused by LC3-associated phagocytosis in cirrhosis patients [19]. Collectively, these recent reports suggest that the intrahepatic macrophage phenotype shifts from an inflammatory phenotype to an anti-inflammatory or pro-fibrogenic phenotype during the progression of liver fibrosis. In this study, we also demonstrated the upregulation of pro-inflammatory genes in F1/F2 fibrosis and subsequent decrease in the expression of these genes in F3/F4 fibrosis; however, the profibrogenic CHIT1 gene was upregulated in the advanced fibrosis stages. Interestingly, a recent report demonstrated that SPP1^high^ fibrogenic macrophages contribute significantly to lung fibrosis in idiopathic pulmonary fibrosis, and these macrophages express high levels of the CHIT1 gene [20].

In this study, using diseased liver tissues, we identified novel immune-associated gene signatures that can reliably predict advanced fibrosis. The signature is composed of five immune-related genes, namely CHIT1, FCER1G, OSM, VEGFA, and ZAP70. Among these genes, CHIT1 expression increased in F3/4 fibrosis, whereas that of the other genes decreased. CHIT1 is primarily secreted by activated macrophages under both normal and inflammatory conditions [21,22]. In HIV-infected smokers, CHIT1 expression in alveolar macrophages correlates with the expression of genes involved in innate immune responses [23]. In the liver, CHIT1 is primarily produced by activated KCs, which activate hepatic stellate cells to induce liver fibrosis [21,22]. FCER1G, also known as FcRγ, is constitutively expressed by monocytes and macrophages, and its expression can be induced in other myeloid cell types, such as neutrophils and eosinophils, when stimulated with IFN-γ, granulocyte colony-stimulating factor, IFN-α, and IL-12 [24]. OSM, VEGFA, and ZAP70 are also associated with the proinflammatory phenotype of macrophages.

Our meta-data analysis identified two genes associated with the gene signature for the advanced fibrosis: *PIK3R1* and *STAT3*. *PIK3R1* encodes the 85-kDa regulatory subunit of PI3K, p85α [25]. A human immunodeficiency was reported in patients with mutations in the PIK3R1 gene, suggesting that PI3K activity is critical in the activity of T and B cells [26]. Moreover, our group recently demonstrated that PI3K/Akt/mTOR signaling pathway in hepatic stellate cells contributes to the liver fibrogenesis [27]. We demonstrated that tenofovir disoproxil fumarate, an antiviral drug for HBV, directly ameliorates liver fibrosis in mice by downregulating the PI3K/Akt/mTOR signaling pathway, which results in the apoptosis of activated hepatic stellate cells [27]. Therefore, targeting PI3K pathway may be a possible therapeutic option for liver fibrosis.

STAT3 is a critical component of the JAK-STAT signaling pathway; however, deregulated STAT3 signaling results in inflammation, cancer, and fibrosis [28]. In this study, the meta-analyses using expression profiles revealed that PIK3R1 and STAT3 are highly interacting genes with the gene signature. STAT3 is a druggable target, and a recent study demonstrated that pharmacological inhibition of the STAT3 pathway ameliorates acute liver injury in vivo by controlling intrahepatic inflammatory macrophages [29]. Recent reports have indicated that STAT3 and its related cytokines have complex biological effects in liver fibrosis [30]. STAT3 pathway activation principally plays pro-inflammatory roles in liver fibrogenesis [30]. Recent clinical trials have demonstrated that liraglutide or semaglutide is an effective nonalcoholic steatohepatitis treatment [31,32]. A recent study demonstrated that liraglutide treatment decreases hepatic inflammation and injury in a nonalcoholic steatohepatitis animal model [33]. Specifically, liraglutide modulated primary KCs to M2-like activation via the STAT3 signaling pathway in wild mouse livers [34]. In palmitic-acid-treated macrophages, the amount of pSTAT3 decreased after treatment with liraglutide, suggesting that STAT3-mediated proinflammatory signaling in macrophages is critical in nonalcoholic steatohepatitis livers [34].

## 5. Conclusions

In conclusion, using comprehensive expression analyses, we developed a novel immune-related gene signature that reliably predicts advanced fibrosis in chronic liver diseases. This signature revealed STAT3 as a potential therapeutic target for the treatment of liver fibrosis.

## Figures and Tables

**Table 1:** Baseline characteristics of enrolled patients.

	Fibrosis Stage 1–2 (N = 24)		Fibrosis Stage 3–4 (N = 53)		*p* Value
	No.	%	No.	%	
Sex					0.5556 ^
Male	18	75.0	43	81.1	
Female	6	25.0	10	18.9	
Mean age (±SD)	53.3 (±12.3)		51.0 (±7.9)		0.4474 *
Hepatitis B					0.0606 ^
No	8	33.3	7	13.2	
Yes	16	66.7	46	86.8	
Hepatitis C					1.0000 ^
No	24	100.0	51	96.2	
Yes	0	0.0	2	3.8	
Diabetes					0.4761 #
No	17	70.8	43	81.1	
Yes	7	29.2	10	18.9	
BMI					0.2546 #
≤25 kg/m^2^	18	75.0	31	58.5	
>25 kg/m^2^	6	25.0	22	41.5	
ALT					0.7843 #
<31 (F), <41 (M) IU/L	16	66.7	32	60.4	
≥31 (F), ≥41 (M) IU/L	8	33.3	21	39.6	
AST					0.2101 #
<31 (F), <37 (M) IU/L	10	41.7	13	24.5	
≥31 (F), ≥37 (M) IU/L	14	58.3	40	75.5	
Platelets					0.0144 #
<150 × 10^9^/L	4	16.7	26	49.1	
≥150 × 10^9^/L	20	83.3	27	50.9	
Antiviral treatment					0.0143 ^
No	24	100.0	42	79.2	
Yes	0	0.0	11	20.8	
Fibrosis					<0.0001 ^
Stage 1	12	50.0	0	0.0	
Stage 2	12	50.0	0	0.0	
Stage 3	0	0.0	25	47.2	
Stage 4	0	0.0	28	52.8	
Etiology					0.0543 ^
HBV	16	66.6	46	86.8	
HCV	0	0.0	2	3.8	
Alcohol	4	16.7	3	5.6	
Others	4	16.7	2	3.8	

SD, standard deviation; BMI, body mass index; ALT, alanine aminotransferase; AST, aspartate aminotransferase. # Chi-squared test; ^ Fisher’s exact test; * Wilcoxon rank-sum test.

**Table 2:** Gene signatures derived from the expression analysis.

Rank	Gene Signature	No. of Gene	Logistic Regression Continuous *p*-Value	ROC AUC	Threshold	Sensitivity	Specificity	Accuracy	PPV	NPV
1	CHIT1_FCER1G_OSM_VEGFA_ZAP70	5	3.45 × 10^−4^	0.945	−11.152614	96.23	91.67	94.81	96.23	91.67
2	FCER1G_LTB_MME_OSM_VEGFA	5	9.21 × 10^−4^	0.954	−11.766822	94.34	91.67	93.51	96.15	88.00
3	CD1B_CHIT1_FCER1G_OSM_VEGFA	5	6.44 × 10^−4^	0.951	−9.544123	94.34	91.67	93.51	96.15	88.00
4	HLADRB3_OSM_VEGFA_ZAP70	4	4.05 × 10^−4^	0.951	−11.382897	94.34	91.67	93.51	96.15	88.00
5	CHIT1_FCER1G_MAF_OSM_VEGFA	5	2.08 × 10^−4^	0.950	−11.392033	94.34	91.67	93.51	96.15	88.00
6	CHIT1_OSM_VEGFA_ZAP70	4	1.01 × 10^−3^	0.950	−8.781110	94.34	91.67	93.51	96.15	88.00
7	CD1B_OSM_TAPBP_VEGFA_ZAP70	5	7.31 × 10^−4^	0.943	−13.930554	94.34	91.67	93.51	96.15	88.00
8	CCL25_CHIT1_OSM_VEGFA_ZAP70	5	7.92 × 10^−4^	0.943	−9.519937	94.34	91.67	93.51	96.15	88.00
9	CLEC4C_OSM_TAPBP_VEGFA_ZAP70	5	5.52 × 10^−4^	0.940	−13.622690	94.34	91.67	93.51	96.15	88.00

ROC, receiver operating characteristic; AUC, area under curve; PPV, positive predictive value; NPV, negative predictive value; CHIT1, chitinase1; FCER1G, Fc fragment of IgE receptor Ig; OSM, oncostatin M; VEGFA, vascular endothelial growth factor A; ZAP70, zeta chain of T-cell receptor associated protein kinase 70; LTB, lymphotoxin beta; MME, membrane metalloendopeptidase; CD1B, CD1b molecule; HLADRB3, major histocompatibility complex, class II, DR beta 3; MAF, MAF bZIP transcription factor; TAPBP, TAP binding protein; CCL25, C-C motif chemokine ligand 25; CLEC4C, C-type lectin domain family 4 member C.

**Table 3:** Uni/Multi-variable logistic regression analysis.

Univariable Logistic Regression						
Variable	n	Coefficient	Odds Ratio (95% CI)	se (Coefficient)	z	*p*-Value
Gene signature (Low vs. High)	77	5.6366	280.50 (37.11–2120.26)	1.0320	5.462	4.72 × 10^−8^
Age (≤55 years vs. >55 years)	77	−0.2364	0.79 (0.28–2.23)	0.5296	−0.446	0.655
Sex (Male vs. Female)	77	−0.3600	0.70 (0.22–2.21)	0.5878	−0.612	0.540
HBV (absent vs. present)	77	1.1896	3.29 (1.03–10.51)	0.5934	2.005	4.50 × 10^−2^
BMI (≤25 kg/m^2^ vs. >25 kg/m^2^)	77	0.7557	2.13 (0.73–6.23)	0.5477	1.380	0.168
Diabetes (absent vs. present)	77	−0.5713	0.56 (0.18–1.73)	0.5700	−1.002	0.316
ALT (<31(F), <41(M) IU/L vs. ≥31(F), ≥41(M) IU/L)	77	0.2719	1.31 (0.48–3.61)	0.5161	0.527	0.598
AST (<31(F), <37(M) IU/L vs. ≥31(F), ≥37(M) IU/L)	77	0.7875	2.20 (0.79–6.12)	0.5228	1.506	0.132
Albumin (<4.0 g/dL vs. ≥4.0 g/dL)	77	0.3314	1.39 (0.49–3.96)	0.5335	0.621	0.534
Platelets (<150 × 10^9^/L vs. ≥150 × 10^9^/L)	77	−1.5717	0.21 (0.06–0.69)	0.6128	−2.565	1.03 × 10^−2^
Antiviral treatment (absent vs. present)	77	18.0065	66085024.42 (0.00–Inf)	1966.6495	0.009	0.993
Simple steatosis (absent vs. present)	77	−035477	0.58 (0.16–2.05)	0.6459	−0.848	0.396
Multivariable logistic regression						
Variable	n	coefficient	Odds ratio (95% CI)	se (coefficient)	z	*p*-value
Gene signature (low vs. high)	77	6.2559	521.08 (31.21–8700.42)	1.4364	4.355	1.33 × 10^−5^
HBV (absent vs. present)	77	2.1565	8.64 (0.59-126.12)	1.3678	1.577	0.115
Platelets (<150 × 10^9^/L vs. ≥150 × 10^9^/L)	77	0.5786	1.78 (0.13–24.46)	1.3359	0.433	0.665

**Table 4:** Meta-data analysis.

High Interaction Pathways	High Interaction Genes
Kaposi sarcoma-associated herpesvirus infection Human papillomavirus infection Epstein–Barr virus infection	PIK3R1 STAT3

PIK3R1, phosphoinositide-3-kinase regulatory subunit 1; STAT3, signal transducer and activator of transcription 3.

## Supplementary Materials

The following supporting information can be downloaded at: https://www.mdpi.com/article/10.3390/biomedicines10010180/s1, Figure S1: Expression of each gene comprising the gene signature in each stage of liver fibrosis, Figure S2: Expression of each gene comprising the gene signature in F0, F1/2, and F3/4.

## Abbreviations

CHIT1: Chitotriosidase, HCC: hepatocellular carcinoma, KCs: Kupffer cells, MoMfs: monocyte-derived macrophages, ROC: receiver operating characteristic

## References

[1] Lesmana C.R.A., Raharjo M., Gani R.A. (2020). Managing liver cirrhotic complications: Overview of esophageal and gastric varices. Clin. Mol. Hepatol..

[2] Odagiri N., Matsubara T., Sato-Matsubara M., Fujii H., Enomoto M., Kawada N. (2020). Anti-fibrotic treatments for chronic liver diseases: The present and the future. Clin. Mol. Hepatol..

[3] Hoshida Y., Villanueva A., Sangiovanni A., Sole M., Hur C., Andersson K.L., Chung R.T., Gould J., Kojima K., Gupta S. (2013). Prognostic gene expression signature for patients with hepatitis C-related early-stage cirrhosis. Gastroenterology.

[4] Goytain A., Ng T. (2020). NanoString nCounter Technology: High-Throughput RNA Validation. Methods Mol. Biol..

[5] Park I.J., Yu Y.S., Mustafa B., Park J.Y., Seo Y.B., Kim G.D., Kim J., Kim C.M., Noh H.D., Hong S.M. (2020). A Nine-Gene Signature for Predicting the Response to Preoperative Chemoradiotherapy in Patients with Locally Advanced Rectal Cancer. Cancers.

[6] Sung P.S. (2021). Crosstalk between tumor-associated macrophages and neighboring cells in hepatocellular carcinoma. Clin. Mol. Hepatol..

[7] Gomez Perdiguero E., Klapproth K., Schulz C., Busch K., Azzoni E., Crozet L., Garner H., Trouillet C., de Bruijn M.F., Geissmann F. (2015). Tissue-resident macrophages originate from yolk-sac-derived erythro-myeloid progenitors. Nature.

[8] Tacke F., Zimmermann H.W. (2014). Macrophage heterogeneity in liver injury and fibrosis. J. Hepatol..

[9] Carpino G., Del Ben M., Pastori D., Carnevale R., Baratta F., Overi D., Francis H., Cardinale V., Onori P., Safarikia S. (2020). Increased Liver Localization of Lipopolysaccharides in Human and Experimental NAFLD. Hepatology.

[10] Ruf B., Heinrich B., Greten T.F. (2021). Immunobiology and immunotherapy of HCC: Spotlight on innate and innate-like immune cells. Cell Mol. Immunol..

[11] Ramachandran P., Dobie R., Wilson-Kanamori J.R., Dora E.F., Henderson B.E.P., Luu N.T., Portman J.R., Matchett K.P., Brice M., Marwick J.A. (2019). Resolving the fibrotic niche of human liver cirrhosis at single-cell level. Nature.

[12] Kim J., Hong S.J., Park J.Y., Park J.H., Yu Y.S., Park S.Y., Lim E.K., Choi K.Y., Lee E.K., Paik S.S. (2010). Epithelial-mesenchymal transition gene signature to predict clinical outcome of hepatocellular carcinoma. Cancer Sci..

[13] Sanyal A.J., Van Natta M.L., Clark J., Neuschwander-Tetri B.A., Diehl A., Dasarathy S., Loomba R., Chalasani N., Kowdley K., Hameed B. (2021). Prospective Study of Outcomes in Adults with Nonalcoholic Fatty Liver Disease. N. Engl. J. Med..

[14] Wen Y., Lambrecht J., Ju C., Tacke F. (2021). Hepatic macrophages in liver homeostasis and diseases-diversity, plasticity and therapeutic opportunities. Cell Mol. Immunol..

[15] Bernsmeier C., van der Merwe S., Perianin A. (2020). Innate immune cells in cirrhosis. J. Hepatol..

[16] Chen Y.Y., Arndtz K., Webb G., Corrigan M., Akiror S., Liaskou E., Woodward P., Adams D.H., Weston C.J., Hirschfield G.M. (2019). Intrahepatic macrophage populations in the pathophysiology of primary sclerosing cholangitis. JHEP Rep..

[17] Cheng D., Chai J., Wang H., Fu L., Peng S., Ni X. (2021). Hepatic macrophages: Key players in the development and progression of liver fibrosis. Liver Int..

[18] Jophlin L.L., Cao S., Shah V.H. (2020). The Transcriptome of Hepatic Fibrosis Revealed by Single-Cell RNA Sequencing. Hepatology.

[19] Wan J., Weiss E., Ben Mkaddem S., Mabire M., Choinier P.M., Picq O., Thibault-Sogorb T., Hegde P., Pishvaie D., Bens M. (2020). LC3-associated phagocytosis protects against inflammation and liver fibrosis via immunoreceptor inhibitory signaling. Sci. Transl. Med..

[20] Morse C., Tabib T., Sembrat J., Buschur K.L., Bittar H.T., Valenzi E., Jiang Y., Kass D.J., Gibson K., Chen W. (2019). Proliferating SPP1/MERTK-expressing macrophages in idiopathic pulmonary fibrosis. Eur. Respir. J..

[21] Kanneganti M., Kamba A., Mizoguchi E. (2012). Role of chitotriosidase (chitinase 1) under normal and disease conditions. J. Epithel. Biol. Pharmacol..

[22] Malaguarnera L., Di Rosa M., Zambito A.M., dell’Ombra N., Di Marco R., Malaguarnera M. (2006). Potential role of chitotriosidase gene in nonalcoholic fatty liver disease evolution. Am. J. Gastroenterol..

[23] Logue E.C., Neff C.P., Mack D.G., Martin A.K., Fiorillo S., Lavelle J., Vandivier R.W., Campbell T.B., Palmer B.E., Fontenot A.P. (2019). Upregulation of Chitinase 1 in Alveolar Macrophages of HIV-Infected Smokers. J. Immunol..

[24] Bournazos S., Wang T.T., Ravetch J.V. (2016). The Role and Function of Fcgamma Receptors on Myeloid Cells. Microbiol. Spectr..

[25] Vallejo-Diaz J., Chagoyen M., Olazabal-Moran M., Gonzalez-Garcia A., Carrera A.C. (2019). The Opposing Roles of PIK3R1/p85alpha and PIK3R2/p85beta in Cancer. Trends Cancer.

[26] Deau M.C., Heurtier L., Frange P., Suarez F., Bole-Feysot C., Nitschke P., Cavazzana M., Picard C., Durandy A., Fischer A. (2014). A human immunodeficiency caused by mutations in the PIK3R1 gene. J. Clin. Investig..

[27] Lee S.W., Kim S.M., Hur W., Kang B.Y., Lee H.L., Nam H., Yoo S.H., Sung P.S., Kwon J.H., Jang J.W. (2021). Tenofovir disoproxil fumarate directly ameliorates liver fibrosis by inducing hepatic stellate cell apoptosis via downregulation of PI3K/Akt/mTOR signaling pathway. PLoS ONE.

[28] Zhao J., Qi Y.F., Yu Y.R. (2021). STAT3: A key regulator in liver fibrosis. Ann. Hepatol..

[29] Ozturk Akcora B., Vassilios Gabriel A., Ortiz-Perez A., Bansal R. (2020). Pharmacological inhibition of STAT3 pathway ameliorates acute liver injury in vivo via inactivation of inflammatory macrophages and hepatic stellate cells. FASEB Bioadv..

[30] Bharadwaj U., Kasembeli M.M., Robinson P., Tweardy D.J. (2020). Targeting Janus Kinases and Signal Transducer and Activator of Transcription 3 to Treat Inflammation, Fibrosis, and Cancer: Rationale, Progress, and Caution. Pharmacol. Rev..

[31] Newsome P.N., Buchholtz K., Cusi K., Linder M., Okanoue T., Ratziu V., Sanyal A.J., Sejling A.S., Harrison S.A., Investigators N.N. (2021). A Placebo-Controlled Trial of Subcutaneous Semaglutide in Nonalcoholic Steatohepatitis. N. Engl. J. Med..

[32] Armstrong M.J., Gaunt P., Aithal G.P., Barton D., Hull D., Parker R., Hazlehurst J.M., Guo K., Abouda G., LEAN trial team (2016). Liraglutide safety and efficacy in patients with non-alcoholic steatohepatitis (LEAN): A multicentre, double-blind, randomised, placebo-controlled phase 2 study. Lancet.

[33] Ipsen D.H., Rolin B., Rakipovski G., Skovsted G.F., Madsen A., Kolstrup S., Schou-Pedersen A.M., Skat-Rordam J., Lykkesfeldt J., Tveden-Nyborg P. (2018). Liraglutide Decreases Hepatic Inflammation and Injury in Advanced Lean Non-Alcoholic Steatohepatitis. Basic Clin. Pharmacol. Toxicol..

[34] Li Z., Feng P.P., Zhao Z.B., Zhu W., Gong J.P., Du H.M. (2019). Liraglutide protects against inflammatory stress in non-alcoholic fatty liver by modulating Kupffer cells M2 polarization via cAMP-PKA-STAT3 signaling pathway. Biochem. Biophys. Res. Commun..

