# Supplementary material for: A Unique Immune-Related Gene Signature Represents Advanced Liver Fibrosis and Reveals Potential Therapeutic Targets
Source: Biomedicines. 2022 Jan 16;10(1):180. doi: 10.3390/biomedicines10010180 (PMC8774116; doi:10.3390/biomedicines10010180)
Supplement: Supplementary file 1 [file biomedicines-10-00180-s001.zip › biomedicines-1546260-supplementary.pdf]

Supplementary Figure 1

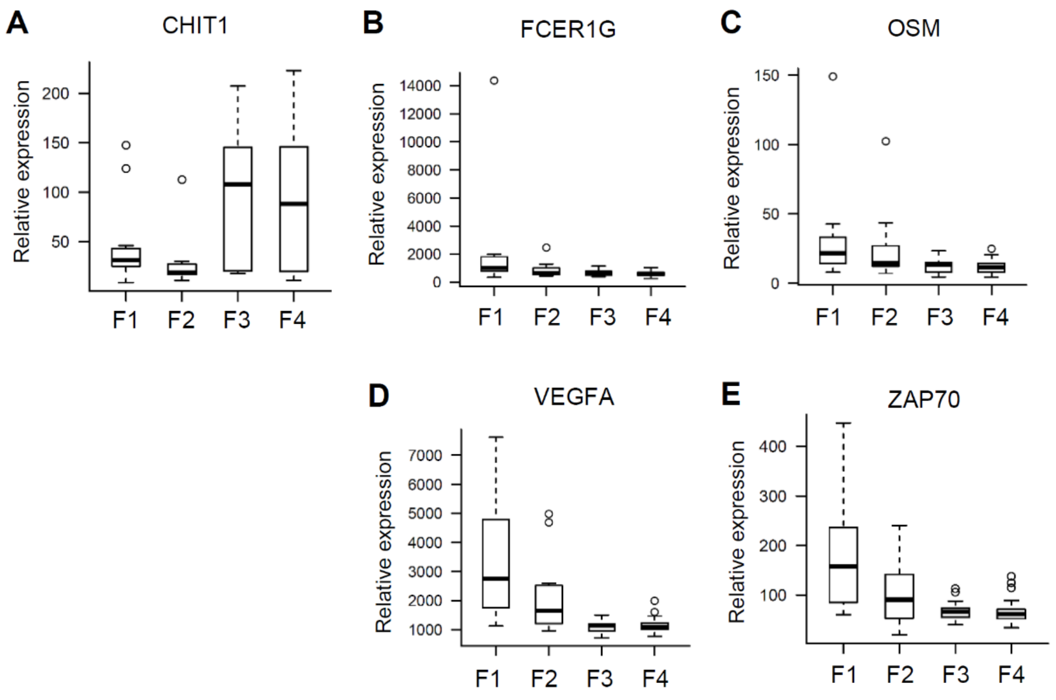

Expression of each gene comprising the gene signature in each stage of liver fibrosis. Fibrosis stage 1 (n = 12), stage 2 (n = 12), stage 3 (n = 25), and stage 4 (n = 28). (A) CHIT1. (B) FCER1G. (C) OSM. (D) VEGFA. (E) ZAP70.

Supplementary Figure 2

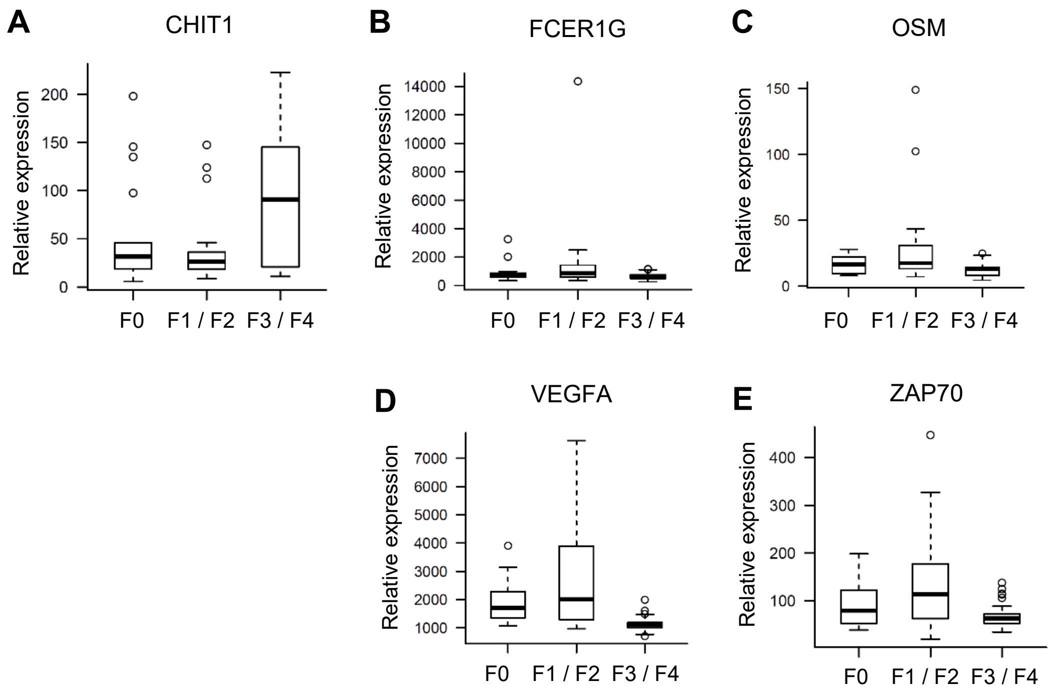

Expression of each gene comprising the gene signature in F0, F1/2, and F3/4. (A) CHIT1. (B) FCER1G. (C) OSM. (D) VEGFA. (E) ZAP70.
